# Effect of Polymer Matrix on Inelastic Strain Development in PI- and PEI-Based Composites Reinforced with Short Carbon Fibers under Low-Cyclic Fatigue

**DOI:** 10.3390/polym15051228

**Published:** 2023-02-28

**Authors:** Sergey V. Panin, Alexey A. Bogdanov, Alexander V. Eremin, Dmitry G. Buslovich, Ivan S. Shilko

**Affiliations:** 1 Laboratory of Mechanics of Polymer Composite Materials, Institute of Strength Physics and Materials Science of Siberian Branch of Russian Academy of Sciences, 634055 Tomsk, Russia; 2 Department of Materials Science, Engineering School of Advanced Manufacturing Technologies, National Research Tomsk Polytechnic University, 634050 Tomsk, Russia; 3 Laboratory of Nanobioengineering, Institute of Strength Physics and Materials Science of Siberian Branch of Russian Academy of Sciences, 634055 Tomsk, Russia; 4 Metal-Polymer Research Institute of National Academy of Sciences of Belarus, 246050 Gomel, Belarus

**Keywords:** short carbon fibers, particulate composite, polyimide, polyetherimide, low-cycle fatigue, adhesion, inelastic strain, mechanical hysteresis loop, High-Performance Polymers

## Abstract

Since the inelastic strain development plays an important role in the low-cycle fatigue (LCF) of High-Performance Polymers (HPPs), the goal of the research was to study the effect of an amorphous polymer matrix type on the resistance to cyclic loading for both polyimide (PI)- and polyetherimide (PEI)-based composites, identically loaded with short carbon fibers (SCFs) of various lengths, in the LCF mode. The fracture of the PI and PEI, as well as their particulate composites loaded with SCFs at an aspect ratio (AR) of 10, occurred with a significant role played by cyclic creep processes. Unlike PEI, PI was less prone to the development of creep processes, probably because of the greater rigidity of the polymer molecules. This increased the stage duration of the accumulation of scattered damage in the PI-based composites loaded with SCFs at AR = 20 and AR = 200, causing their greater cyclic durability. In the case of SCFs 2000 µm long, the length of the SCFs was comparable to the specimen thickness, causing the formation of a spatial framework of unattached SCFs at AR = 200. The higher rigidity of the PI polymer matrix provided more effective resistance to the accumulation of scattered damage with the simultaneously higher fatigue creep resistance. Under such conditions, the adhesion factor exerted a lesser effect. As shown, the fatigue life of the composites was determined both by the chemical structure of the polymer matrix and the offset yield stresses. The essential role of the cyclic damage accumulation in both neat PI and PEI, as well as their composites reinforced with SCFs, was confirmed by the results of XRD spectra analysis. The research holds the potential to solve problems related to the fatigue life monitoring of particulate polymer composites.

## 1. Introduction

Recently, interest in High-Performance Polymers (HPPs) has grown significantly in advanced industries due to the possibility of designing composites with unique performance properties [1,2]. Polyimide (PI) belongs to the HPP class and, because of its chemical structure, is one of the most temperature-resistant high-strength amorphous thermoplastics (at the top of the pyramid of polymer structural materials [3]). Typically, its strength characteristics are improved by loading with reinforcing fibers and corresponding dispersion hardening or by continuous fiber reinforcement.

Both the low melt flow index of PI and its poor processability result in the development of polyetherimide (PEI) for some industrial applications at relatively low operating temperatures [4]. The incorporation of ether bonds into a polyimide molecular chain contributes to the flexibility required for satisfactory processability by conventional production routes. At the same time, the excellent PEI mechanical properties, almost as great as those for the PI, are retained. This fact caused a large number of PEI-based composites to appear on the market for various applications, determined primarily by both the types and contents of reinforcing fillers. Thus, cost, processability, and temperature stability are becoming the key factors in terms of choosing a particular type (grade) of commercially available composite materials [5].

As known, continuous-fiber-reinforced composites (laminates) possess the greatest physical and mechanical characteristics. However, their production routes are complex and difficult to automate [6]. On the other hand, particulate composites have less strength but can be effectively processed by some methods, conventional for thermoplastics [7]. Consequently, their manufacturing is significantly cheaper and more technologically advanced due to using the composites as feedstock for the fabrication of products.

When designing polymer composites for structural applications (without special requirements for additional functional properties, such as thermal/electrophysical, antifriction, biological, etc.), thermoplastic materials are typically highly loaded with short synthetic fibers (about 20–40 wt.%). This provides multiple increases in their elastic modulus, ultimate tensile strength, and offset yield stress, but reduces strain at failure. Unlike laminates, particulate composites are designed to resist loads comparable to the bearing capacity of the polymer matrix. With an increase in operational loads, due to the multiple differences in the elastic moduli of the components, intense strain processes develop at their numerous interfaces, culminating in local fractures. The matrix role is reduced not only to ensure the adhesive interaction with fillers but also significantly affects the mechanical properties of the composites. This is greatly determined by both molecular structure and crystallinity degree.

M. Zaghloul et al. [8] reviewed the effect of nanofillers of various kinds and sizes on the mechanical properties of polyester-based composites. The maximum increase in the properties was revealed when nanofiller and fibers were added simultaneously. Another paper by the same authors [9] addressed the effect of stress level as well as the weight fraction of glass fibers on the fatigue life of polyester-based composites. It was shown that the failure mechanism depended on the level of applied stress; under high stress, the fracture was induced by plastic deformation, while at lower stress, it was induced by damage accumulation. M. Zaghloul et al. [10] showed that the enforcement pattern could vary the fatigue life of glass-fiber-reinforced polyester-based composites by 61 times. The prospects of employing X-ray diffraction (XRD), Fourier transform infrared reflectance (FTIR), transmission electron microscopy (TEM), and a zeta-sizer analyzer for fiber-reinforced thermosetting composites embedded with nanoparticles fatigue analysis were illustrated in [11]. The results of studies on the mechanical properties of highly filled polypropylene composites might be found elsewhere in [12,13,14].

Xie et al. [15,16] studied the effect of the aspect ratio (AR) of graphene oxide on the mechanical properties of polypropylene-based composites. The results showed that the increase in AR up to 3000 times gave rise to a certain improvement of the mechanical characteristics, while with further saturation, no improvement was registered. A. Shchegolkov et al. [17] studied the regularities of heat generation under the mechanical deformation of elastomers modified by loading with multiwall carbon nanotubes (MW CNT). As shown, the intensity of heat release was revealed with the increase in the MW CNT volume fraction.

A large number of papers address the structure and, mainly, tribological properties of both PI and PEI as well as their composites. For example, varying lengths of carbon fibers (CFs) [18] showed that loading PI with CFs ~100 µm long gives a lower wear rate than those for both values of 300 and 1000 µm due to better adhesion with the matrix. The treatment of the CFs in a solution of HNO_3_ with diamine increased the adhesion between the filler and the polymer, improving the mechanical properties of the composite [19]. In addition, the effect of temperature on some properties of the PI-based composites reinforced with CFs was described [20,21]. In addition, various modifications of the CFs to enhance the adhesion were suggested [22,23,24].

A significant amount of research is devoted to the analysis of various PEI characteristics. In particular, the onset of the non-linear viscoelastic behavior of PEI-based composites with different contents of the short CFs (SCFs) has been reported [25], which corresponds to 30, 35, 50, and 60 MPa after loading with 0, 5, 10, 20, and 30 vol.% SCFs, respectively. Lee, E. Seok et al.. showed a significant increase in tensile strength [26] from 104 up to 168 MPa after filling with 30 vol.% untreated SCFs and up to 239 MPa after loading with 30 vol.% SCFs treated by hydrogen plasma. Sun, Z. et al.. [27] stated that the mechanical properties of PEI-based composites reinforced with CFs decrease with rising temperature due to poorer contact at the fiber–matrix interface. This was caused by their different thermal expansion coefficients; even finishing with a graphene oxide does not enable the avoidance of such negative effects. Yongxin, P. [28] and Zhang, Y.Y. [29] studied in detail the tribological properties of both neat PEI and its composites reinforced with CFs.

Most of the papers in which authors reported the mechanical properties were focused on tensile strength and wear resistance. Nevertheless, there are very few studies on the fatigue life of particulate PI- and PEI-based composites, although most parts made from them are undoubtedly subjected to cyclic loading.

The fatigue properties of both the PI and PEI were partially analyzed by their manufacturers. For the low-cycle fatigue (LCF) mode at a load per cycle of ~74 MPa, the durability of PEI is 10^3^ cycles, but loading with 10% short glass fibers (SGFs) improves this parameter up to 8⋅10^3^ cycles [30]. The length of SGFs greatly affects the fatigue life: the PEI-based composite, loaded with 20% SGFs withstands 2⋅10^4^ cycles, while a similar composite filled with the same chopped SGFs provides only 6⋅10^2^ cycles. A further increase in the content of SGFs of up to 30% in PEI enhances the durability by up to 2⋅10^5^ cycles. In contrast, loading with the same chopped SGFs, as filler, results in only 4⋅10^2^ cycles. Raising the content of SGFs up to 40% no longer increases the durability, which remains at the level of 2⋅10^5^ cycles. Note that ductility-enhanced composites are characterized by a lower fatigue life. For example, PEI reinforced with 45% glass/mineral failed after only 10^3^ cycles at 74 MPa (this level corresponds to the offset yield stress of the neat PEI) [31].

In terms of fatigue, polyimides are less studied. As shown in [32], the durability of PI is 10^5^ cycles under symmetrical loads of 48 MPa, while filling it with 15% graphite deteriorated the composite’s fatigue life over the entire temperature range.

Friedrich, K. et al.. [33,34] studied PI-based composites in the form of aligned short-fiber laminates under cyclic loading. It is claimed that the composites reinforced with the aligned discontinuous CFs can be an alternative material to continuous-fiber-reinforced ones when considering their static and fatigue properties. Compared to polyethersulfone, PI’s properties are slightly lower, but the thermal stability is higher. At a temperature of 100 °C, a negligible decrease in the fatigue life of a reinforced PI-based composite occurred in the LCF range, while its reduction was much greater in the high-cycle fatigue (HCF) mode.

Ansari, M.T.A. et al.. [35] and Mortazavian, S. et al.. [36] investigated the fatigue life aspects for the composites based on thermoplastic binders (matrices). From the standpoint of applying methods for evaluating the behavior of such composites in the fatigue tests, the following approaches were used:

Stiffness reduction [37], according to different scenarios depending on the elastic properties of fibers, the interfacial adhesion levels, or the matrix properties; at the same time, the dynamic and secant elastic moduli are often evaluated [38,39,40,41];

- The recording of acoustic emission signals, for example, to detect the initiation of cracks [42] or evaluate their subsequent propagation;
- The thermal (infrared) imaging inspection of polymers [43,44], applying the measurements’ data to calculate temperature harmonics or solve differential equations of thermodynamics.

In the fatigue behavior of the composites, an important aspect is the polymer structure, the adhesion of reinforcing fibers and matrices, as well as the length and aspect ratio (AR) of the fibers. All of them, among other factors, affect fatigue resistance.

It is reasonable to study the above processes on related thermoplastic polymers, including PI and PEI. In this way, the research aim was to study the effect of the (amorphous) polymer matrix type on the resistance to cyclic loading for both PI- and PEI-based composites, identically loaded with SCFs of various lengths, in the LCF mode (when the inelastic strain development plays a crucial part).

The structure of this paper is presented as follows: Section 2 describes the materials and methods; Section 3.1 reports the data on the structural nature of the composites; Section 3.2 gives a brief description of the Fourier transform infrared (FTIR), Raman, and X-ray diffraction (XRD) spectra for the investigated specimens; Section 3.3 characterizes the physical and mechanical properties of the composites under static tensile conditions; Section 3.4 describes the results of the fatigue tests; Section 4 discusses the summarized data; and Section 5 introduces the conclusions.

## 2. Materials and Methods

**Figure 1:**
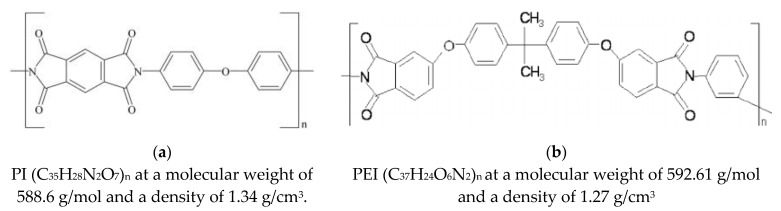
Typical structures of PI (**a**) and PEI (**b**) molecules [4].

Polyimide and polyetherimide in the form of powders were used as the matrix for the composites, namely, the PI (PI-1600, Solver, Jiande, China) and PEI (ULTEM R00H, Solver, Jiande, China). The average particle sizes were 20 and 16 µm for the PI and PEI powders, respectively. The key physical and chemical properties of PI and PEI are presented in Table 1. Figure 1 shows the PI and PEI molecular structure (according to the published data).

The content of SCFs, belonging to the carbonized class, was 10 wt.% in all composites. The characteristics of the fillers are presented in Table 2; their SEM micrographs are shown in the Supplementary Materials in Figure S1. 

The polymer powders and fillers were dispersed and mixed in a suspension of ethyl alcohol using a “PSB-Gals 1335-05” (ultrasonic bath, PSB-Gals Center for Ultrasonic Equipment, Moscow, Russia). The processing time was 3 minutes and the generator frequency was 22 kHz. Then, the suspension was dried in a forced ventilation oven at a temperature of 120 °C for 3 hours. The plates with dimensions of 70 × 60 × 10 mm were made by compression sintering at a pressure of 15 MPa and a temperature of 370 °C, then cooling at a rate of 2 °C/min. The specimens were cut from the plates by using a CNC milling machine. The required surface quality of the specimens was achieved by grinding with sandpapers up to P2000 (ISO 6344).

Both static tensile and fatigue tests were carried out on dog-bone type V specimens, according to ASTM D638 and D7791, using a Biss Nano 15 kN (servo-hydraulic machine, BISS, Bangalore, India). The length of the specimens was 64 mm, the gauge length was 10 mm, and the cross-sectional area was 3.2 × 3.2 mm. The strains were measured by a non-contact (optical) method by using a “VIC 3D” setup (Digital Image Correlation (DIC) method, Correlated Solutions, Columbia, SC, USA). The static tests were conducted at a loading (cross-head) speed of 1 mm/min, according to ASTM D638, recording the load–extension curve of the specimen. The fatigue tests were carried out for all materials in the load control at a maximum stress per cycle of 74 MPa, recording the time–load and time–strain dependences. The cycle asymmetry coefficient was R = 0. This provided the LCF mode for all studied neat polymers and composites. In order to exclude the influence of possible heating on the deformation behavior, the frequency of cyclic loading did not exceed 1 Hz. The pulse shape was sinusoidal.

In order to quantify the mechanical behavior and changes in the material properties, the following parameters of mechanical hysteresis loops were determined: area and both secant and dynamic moduli [45]. The loop area was assessed as the difference between the integrals of its two parts, reflecting the loading and unloading half-periods, respectively:

(1) $$S=\overset{\delta_{max}}{\underset{\delta_{min}}{\int }}\sigma \left(\delta \right)d\delta -\overset{\delta_{min}}{\underset{\delta_{max}}{\int }}\sigma \left(\delta \right)d\delta$$

where *δ_min_* and *δ_max_* are the strain levels corresponding to the beginning and end of a cycle.

The dynamic and secant moduli of elasticity were determined as follows:

(2) $$E_{dyn}=\frac{\sigma_{max}\left(\delta_{max}\right)-\sigma_{min}\left(\delta_{min}\right)}{\delta_{max}-\delta_{min}};E_{sec}=\frac{\sigma \left(\delta_{max}\right)}{\delta_{max}}$$

The DIC method was applied to measure the strain in the fatigue tests and draw the hysteresis loops. The proportion of inelastic residual strain, developed in the specimens, was assessed at the minimum load point of the hysteresis loops. Since the cycle asymmetry coefficient was zero, the minimum load per cycle was 0 N as well.

The structural studies were carried out on the cleavage surfaces after a mechanical fracture of the samples with a notch, exposed in liquid nitrogen at a temperature of −197 °C for one hour. The cleavage surfaces were used to characterize the distribution of SCFs and evaluate the overall structure of the composites. A copper film about 10 nm thick was deposited onto the cleavage surfaces using a JEOL JEE-420 (vacuum evaporator, JEOL USA Inc., Peabody, Massachusetts, USA). Their micrographs were taken by using a LEO EVO 50 (scanning electron microscope (SEM), Carl Zeiss, Oberkochen, Germany) at an accelerating voltage of 20 kV.

The IRFS analysis was carried out by attenuating the total internal reflection (ATR) spectroscopy. The ATR spectra were recorded with a Nicolet 5700 (FTIR-Fourier spectrometer, Thermo Fisher Scientific Inc., Waltham, MA, USA) equipped with a single ATR attachment on a Ge crystal in a range of 4000–650 cm^−1^ with a resolution of 4 cm^−1^ and a number of scans of 64. Raman spectra were registered using the same spectrometer and a Raman module. The exciting laser wavelength was 1064 nm, and the number of scans was 64.

XRD investigations were conducted with a Shimadzu XRD-6000 (X-ray diffractometer, Shimadzu, Tokyo, Japan) for additional structural examinations in an angle range of 10–80° with a step of 0.02° and an exposure time of 1 s.

## 3. Results

### 3.1. Scanning Electron Microscopy

Since the strain behavior under both static and cyclic loading is determined by the structure of the reinforced polymer composites, its study was carried out by SEM. Typically, the structure of particulate composites was analyzed at two levels: (i) the formation of interfacial bonds at the “matrix-filler” interface, and (ii) the pattern of the fibers’ distribution in the polymer matrix. In the case of SCFs at AR = 10, the filler could be considered as elongated particles (particle fibers), including from the viewpoint of their predominant orientation. At AR = 200, the length of SCFs became comparable to the specimen dimensions; for example, their thickness. In this case, their orientation was critical from the standpoint of the location of SCFs relative to the applied load axis. Meanwhile, there was a technological issue of ensuring both uniform and multidirectional orientations of SCFs. This was related to the fact that SCFs were predominantly laid perpendicular to the compression axis in the hot sintering process. In addition, it was difficult to avoid both the agglomeration and clumping of SCFs (especially when using a high-speed homogenizer). Finally, the molten polymer was not always able to penetrate into interfiber spaces at high AR values, which could cause the inhomogeneity of the structure and functional properties, as well as great residual stress. In this case, both the interface and filler distribution could be visualized by SEM.

**Figure 2:**
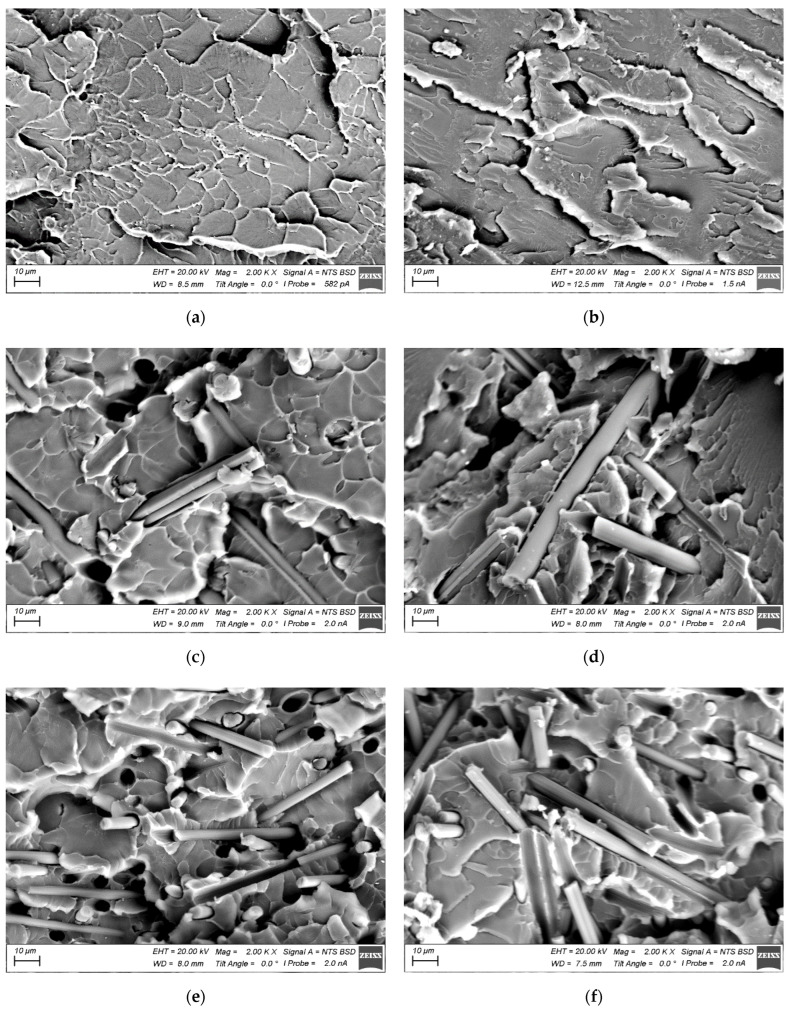

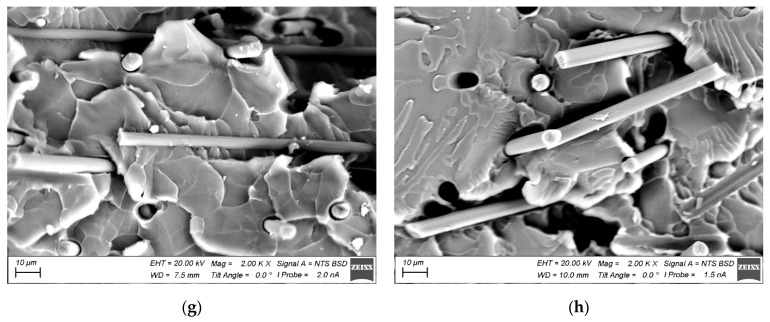
SEM micrographs of the structure of the PI- and PEI-based composites: neat PI (**a**); neat PEI (**b**); PI/CF100 (**c**); PEI/CF100 (**d**); PI/CF200 (**e**); PEI/CF200 (**f**); PI/CF2000 (**g**); PI/CF2000 (**h**).

Figure 2 shows SEM micrographs of the structure of both neat polymers and their composites. The analysis allowed us to conclude that:

1. A “cellular” pattern of the supermolecular structure formed in the neat PI (Figure 2a), whose element sizes were comparable to the particle diameter of the initial PI powder. In the neat PEI, such a “cellular” supermolecular structure was not observed (Figure 2b), although the mechanical properties of both polymers were similar in general (according to the static tensile test data described below).
2. SCFs based on viscose precursor (AR = 10), according to the manufacturer’s data [46], had a high surface area (up to 100 mm/g in the upper limit). However, it did not provide the perfect adhesion between the components of both the PI- (Figure 2c) and PEI-based (Figure 2d) composites.
3. After loading both the PI and PEI with SCFs, the polymer matrix morphology remained similar to that of neat polymers. Simultaneously, the polymer wetting of graphitized 200 (AR = 20) and 2000 µm (AR = 200) SCFs was poor (Figure 2e–h).

### 3.2. FTIR, Raman, and XRD Spectra

**Figure 3:**
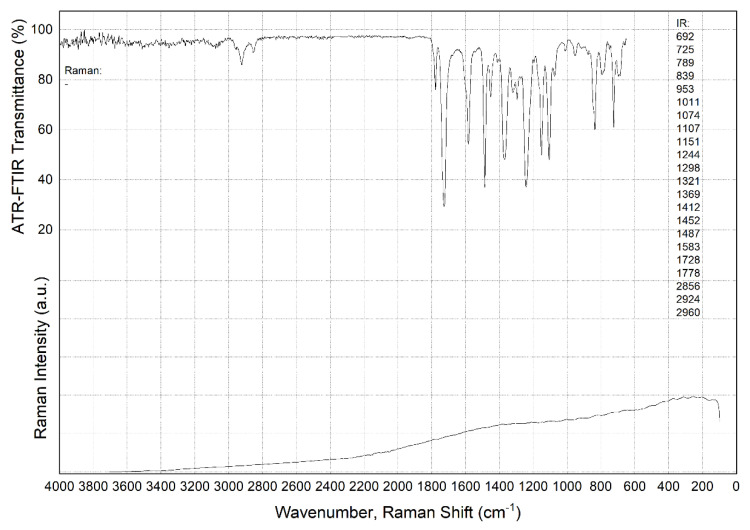
The FTIR and Raman spectra for neat PI.

**Figure 4:**
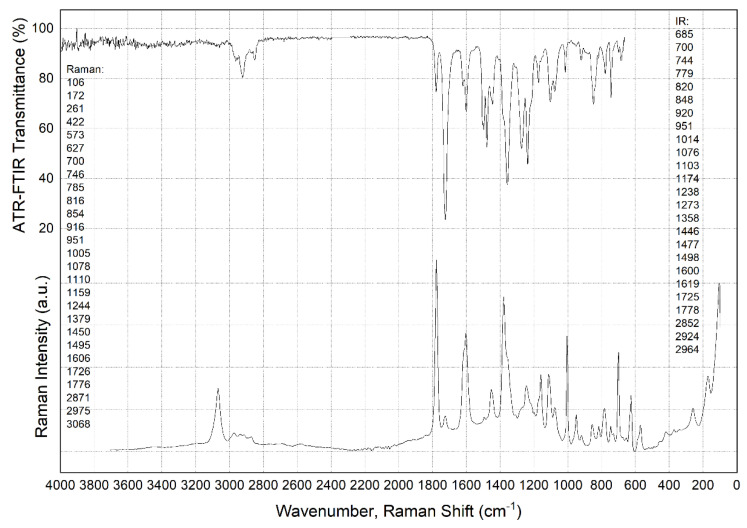
The FTIR and Raman spectra for the neat PEI.

Figure 3 and Figure 4 presents FTIR and Raman spectra showing characteristic peaks for the neat PI and PEI, respectively. Despite the differences in the individual ones [47], the FTIR spectra were similar for the polymers.

Raman spectra analysis also revealed a significant number of characteristic peaks in neat PEI, whose wavelength reciprocals are shown in Figure 4, close to the left ordinate axis. In this research, the authors do not provide a detailed description. More important is that the Raman spectrum for neat PI showed no peaks at all (Figure 3, column next to the left abscissa axis). The reason was the fact that, according to the published data, Raman spectroscopy is sensitive to the symmetric vibrations of nonpolar groups, while FTIR spectroscopy, on the contrary, is susceptible to the asymmetric oscillations of polar clusters of molecules [48]. This reliably indicated the difference between the two types of thermoplastic matrices discussed in the section below from the standpoint of molecular chains’ flexibility.

It should be noted that loading with SCFs did not affect the appearance of the FTIR spectra. Such a result was expected, since the presence of SCFs did not change the molecular structure of the polymer matrices and their interaction with the FTIR radiation formed the analyzed spectrum.

In addition, the authors attempted to identify the changes in the spectrum after the fatigue tests. It was suggested that breaking a number of bonds had to be accompanied by their saturation with the elements from the environment (the appearance of defects during cyclic loading may be accompanied by the oxidation of dangling bonds of the polymers and modify the FTIR spectra). It was expected that this could further characterize some structural changes in the fatigue tests. Selected FTIR spectra are shown in Figure S2 for the PI and Figure S3 for the PEI composites in the Supplementary Section. In the case of the PI/CF100 composite, new peaks were found, but no significant changes and differences were observed on other similar specimens after cyclic loading (the specimen designations are presented in Table 2 below). This fact enabled us to conclude that the measured data for the PI/CF100 composite were somehow externally affected.

### 3.3. Static Tensile Tests

**Figure 5:**
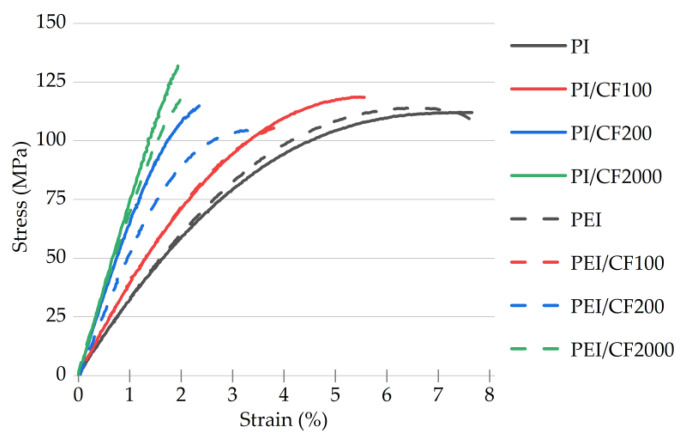
The engineering stress–strain curves for the PI- and PEI-based composites loaded with SCFs of various lengths.

In order to quantify the effect of loading with SCFs of different lengths on the mechanical properties of the composites, static tensile tests were performed. The engineering stress–strain curves are shown in Figure 5; the key mechanical characteristics are presented in Table 3. In addition, for ease of comparison, the last column shows the results of the fatigue tests, presented in detail and discussed below in Section 3.4.

The following patterns should be noted:

1. For the neat polymers, the main characteristics of the PI and PEI were comparable in general. In the PEI case, the elastic modulus of 3.4 GPa was higher by ~180 MPa/5.5%; the strain at a failure value was 1.2 times lower (ε_f_ = 6.6%); and the offset yield stress was higher by 2.5 MPa/4.8% (OYS_0.2_ = 54.9 MPa).
2. Loading with CF100 at AR = 10 (according to Table 2) was accompanied by an increase in both the elastic modulus for both polymers (by 910 and 710 MPa for the PI- and PEI-based composites, respectively) and their offset yield stress. This practically equalized the composites in terms of these parameters. The ultimate tensile strength increased slightly for the PI-based composite but decreased for the PEI-based one. At the same time, the strain at failure values decreased.
3. Loading with CF200 at AR = 20 resulted in enhancing both the elastic modulus and the offset yield stress compared to the neat polymers. For the PI-based composite, a twofold increase in the elastic modulus was observed, while its increase was only 1.6 times for the PEI-based one (up to 6.9 and 5.5 GPa, respectively). The offset yield stress increased by factors of 1.7 and 1.4 for the PI- and PEI-based composites, respectively. As expected, the strain at failure values decreased (down to 2.3% and 3.2%, respectively).
4. The maximum increase in the elastic modulus was for the PI-based composite loaded with CF2000 at AR = 200, which was greater by 1200 MPa than it was in the CF200 case. For the PEI/CF2000 specimen, the elastic modulus differed by 1360 MPa after loading with SCFs 200 and 2000 µm long. The PI/CF2000 composite (121 MPa) had a higher offset yield stress and, accordingly, a lower strain at a failure value of 1.9%.

Thereby, the strength properties expectedly increased with the increasing length of SCFs, regardless of the polymer matrix type, while the strain at failure values decreased. In general, the PEI-based composites were inferior to the PI-based ones in terms of the offset yield stress determined by the matrix properties. Regardless of the length of SCFs, reinforcement was less effective for the PEI-based composites (lower “gain” in properties). These data were used below when analyzing the results of the fatigue tests. The maximum increase in the strength properties (the elastic modulus and the offset yield stresses) up to 2.5 times was after loading with the longest SCFs of 2000 µm.

### 3.4. Fatigue Tests

Based on the above results, as well as from a general understanding of fatigue, it could be assumed that increasing the offset yield stress had to be accompanied by an increase in the number of cycles to failure. In order to test this hypothesis, it was initially required to choose a load in the fatigue tests. As shown in some papers, including the authors’ previous one [49], the influence of the polymer matrix under cyclic loading was most pronounced in the LCF mode. With this fact in mind, as well as on the basis of the results of the static tensile tests, a load level of 74 MPa was chosen as the maximum stress per cycle. This value was characterized by the following patterns:

- It was greater than the OYS_0.2_ levels for both the neat PI and PEI (52.4 and 54.9 MPa, respectively), as well as the composites loaded with CF100 (65.1 and 65.9 MPa, for the PI and PEI matrices, respectively);
- It was comparable to or higher than the OYS_0.2_ levels for the composites filled with CF200 (88 and 75 MPa, for the PI and PEI matrices, respectively);
- It was noticeably lower than after reinforcing with CF2000 (121 and 108 MPa for the PI and PEI composites, respectively).

Thus, the different strain modes were preset for the tested materials at the same load level per cycle: (i) lower, (ii) comparable (equal), and (iii) higher than their offset yield stress. The minimum load per cycle was 0 MPa.

#### 3.4.1. Energy loss due to hysteresis (loop area)

**Figure 6:**
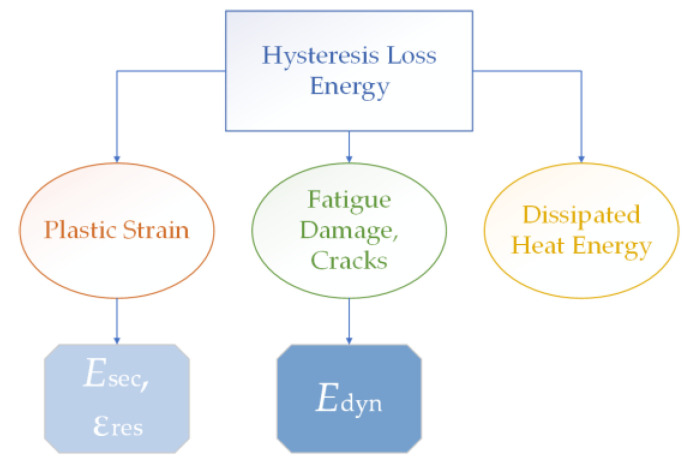
A schematic diagram of the processes composed by the mechanical hysteresis loop.

The loop area *S* reflected the damping properties of the material under cyclic loads, where the amount of unrecovered elastic energy was associated with heat dissipation, damage development, and plastic strain (see the diagram in Figure 6). In the case of the considered amorphous polymers and their composites, the dynamic modulus *E*_dyn_ was a characteristic of a change in stiffness due to the accumulation of scattered damage and microcracks (since the initiation and propagation of the main crack in terms of the hysteresis loops could not be detected and evaluated). According to this mechanism, the fracture occurred during the elastic strain’s development, which is most pronounced in the HCF mode. In turn, the secant modulus *E*_sec_ and residual strain ε_res_ quantitatively characterized elongation (due to a cyclic creep) and typically reflected the specifics of the fracture processes caused by the development of the plastic strains under LCF conditions.

**Figure 7:**
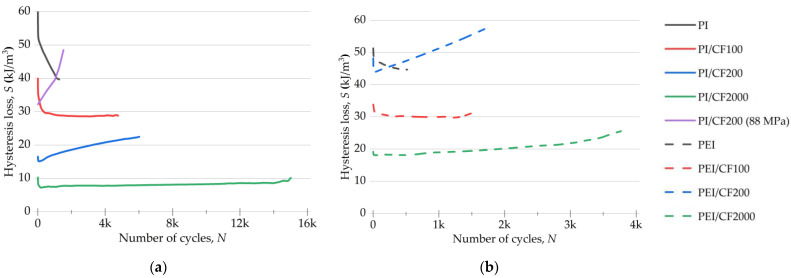
The changes in the loop areas *S* for the PI- (**a**) and PEI-based (**b**) composites under cyclic loads.

Variations in the area of the mechanical hysteresis loops under cyclic loads are shown in Figure 7. At the beginning of the fatigue tests, the hysteresis loss for the neat PI was slightly higher than that for the neat PEI (60 and 50 kJ/m^3^, respectively). Both polymers were characterized by a gradual decrease in their loop area with an increase in the number of cycles. At the same time, the loss decreased to 40 kJ/m^3^ for the neat PI, but it decreased only to 45 kJ/m^3^ in the neat PEI case. For the PI/CF100 and the PEI/CF100 composites (according to Table 2), the *S* values were similar (~30 kJ/m^3^) from the very beginning of the tests, and they remained almost unchanged until the tests were completed.

For the PEI/CF200 composite, the hysteresis loss at the beginning of the test was three times greater than that for the PI/CF200 (44 and 15 kJ/m^3^, respectively) and also exceeded this parameter for the PEI/CF100 (32 kJ/m^3^). Thereby, the losses were associated not only with the offset yield stresses and their ratios with the applied load.

Another distinctive feature of the PI/CF200 and PEI/CF200 composites was the enhancement of this parameter with an increasing number of cycles. At the same time, the losses for the PEI/CF200 exceeded that for the neat PEI by its fracture point (Figure 7b).

Figure 7a also shows a curve for the PI/CF200 composite tested at a load of 88 MPa, close to its offset yield stress. This experiment provided loading conditions similar to those for the PEI/CF200. In this case, the hysteresis loss noticeably increased and sharply rose during the fatigue tests, exceeding that for the neat PI by its fracture point. Such behavior was similar to that of the PEI/CF200 composite, which was also close in terms of durability (1600 cycles to failure).

After loading with the longest SCFs at AR = 200, the hysteresis loss was twice as high for the PEI/CF2000 composite as for the identically filled PI/CF2000 (8 and 18 kJ/m^3^, respectively). As the fatigue tests progressed, the *S* value almost did not change for the PI-based specimen and slightly increased in the PEI-based case. Thus, the mechanical hysteresis loop area was minimal.

In general, there was a linear decrease in the loss rate as the length of SCFs increased for both types of composites under LCF conditions. Both the neat PI and PEI showed almost the same level of hysteresis loss and general behavior in the fatigue tests. Similarly, the PI- and PEI-based composites loaded with CF100 were similar both in initial *S* values and their dynamics. However, in this case, the PEI-based composites filled with PAN SCFs (CF200 and CF2000, according to Table 2) possessed an enhanced level of losses compared to the PI-based ones. Thus, there is a different effect of PAN SCFs (Table 2) on the mechanical behavior of the polymers, which (without fillers) were similar in chemical composition and mechanical properties.

#### 3.4.2. The Secant and Dynamic Modules

**Figure 8:**
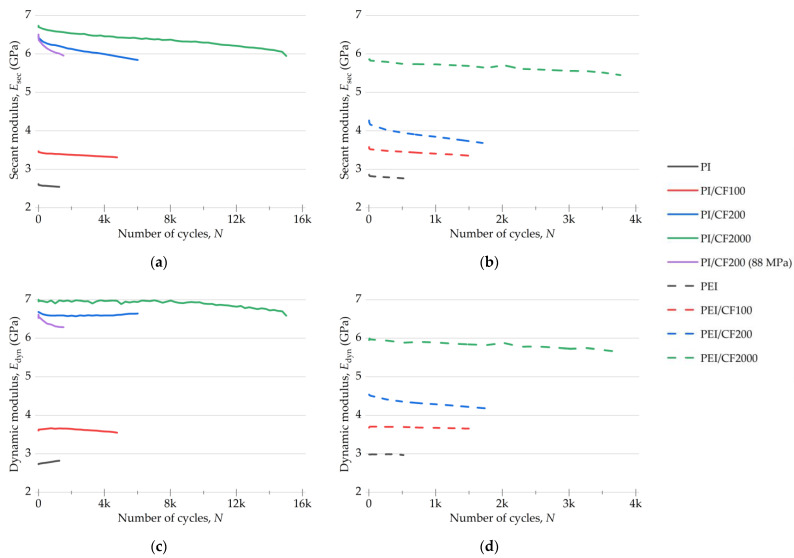
The changes in *E*_sec_ secant (**a**,**b**) and *E*_dyn_ dynamic (**c**,**d**) moduli for the PI- and PEI-based composites under cyclic loads.

**Figure 9:**
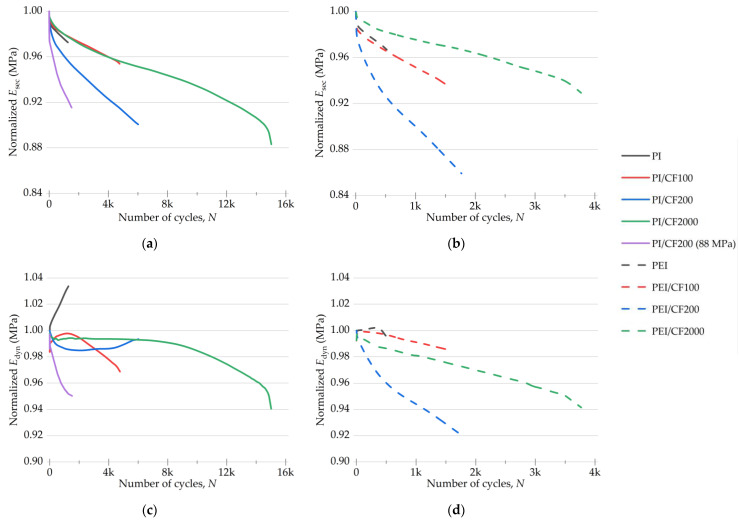
The changes in *E*_sec_ secant (**a**,**b**) and *E*_dyn_ dynamic (**c**,**d**) moduli for the PI- and PEI-based composites under cyclic loads; normalization was made over the initial values.

The plots of the secant and dynamic moduli, calculated from the mechanical hysteresis loops for the PI- and PEI-based composites during the fatigue tests (Figure 8), characterized the changes in their deformation behavior. Figure 9 shows both *E*_sec_ and *E*_dyn_ parameters normalized to their initial values.

A comparison of *E*_sec_ levels for both types of composites (Figure 8) showed that the greatest difference was for the specimens loaded with CF200. The values of both moduli approached the level for the PI/CF2000 one, while both moduli of the PEI/CF200 composite, on the contrary, were closer to those for the PEI/CF100 specimen.

The fatigue test of the PI/CF200 composite at a load of 88 MPa (which was in the order of its offset yield stress) showed that the initial values of both moduli were the same as at a load of 74 MPa. Nevertheless, a sharper decrease in both moduli was observed at 88 MPa. Such behavior for the secant modulus was close, including its level of 9–10%, to that for the PEI/CF200 composite (Figure 9).

At the normalized values, *E*_sec_ reduction was greater for all PEI-based composites compared to the PI-based ones. For both the neat PI and its composites, the secant modulus declined at the same rate, except for the PI/CF200 case at 88 MPa, when the reduction rate was greater. Thus, the highest *E*_sec_ reduction rate was observed for both types of composites loaded with CF200.

The values of the normalized dynamic modulus *E*_dyn_ increased for the neat polymers, which indicated the enhancement of their stiffness and strain hardening under cyclic loading (Figure 9), and decreased for the composites, as a rule. For the PI-based specimens, the decrease did not exceed 6%, but it decreased by 8% in the PEI-based cases.

In general, the variation pattern in the normalized *E*_dyn_ was similar for the composites of both types during their cyclic loading. However, the achievement of the same level occurred later for the PI-based specimens, indicating a slower accumulation of scattered damage.

#### 3.4.3. Residual Strain

**Figure 10:**
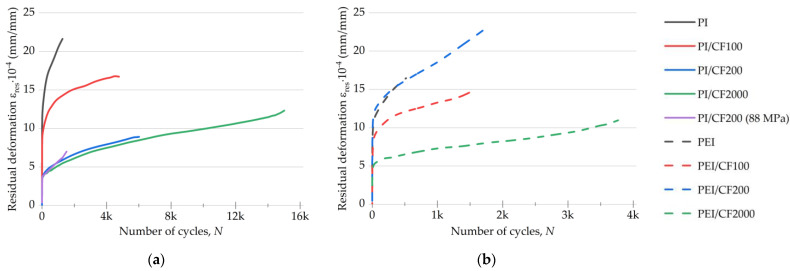
The changes in the residual strain values for the PI- (**a**) and PEI-based (**b**) composites under cyclic loads.

The parameter “residual strains” reflected the tendency of the materials to creep under cyclic loads. The corresponding data for the studied polymers and their composites are given in Figure 10. As expected, loading with SCFs suppressed the development of irreversible strain in the specimens.

In the initial cycles of the fatigue tests, the specimens were sharply elongated (their residual strain enhanced), after which the uniform growth stage began. For the purpose of comparing the composites, further consideration was carried out at 100 loading cycles, at which the residual strain developed uniformly for all studied materials. The following patterns should be noted:

- The residual strain was higher by ~12% for the neat PI than that for the neat PEI (13.9·10^−4^ and 12.4·10^−4^ mm/mm, respectively, according to Figure 10a,b). The increase rates were comparable for both materials (up to ~500 cycles).
- At the beginning of the tests, the residual strain values were close for the PI/CF100 and the PEI/CF100 composites (10.1·10^−4^ and 9.8·10^−4^ mm/mm, respectively). The increase rates for this parameter were also similar.
- For the PEI/CF200 composite, the residual strain level was three times higher than that of the PI/CF200 (4·10^−4^ versus 13·10^−4^ mm/mm). The increase rate was also greater for the PEI/CF200 specimen. At the load stress of 88 MPa, the fatigue test results for the PI/CF200 composite exhibited a similar residual strain value as that at 74 MPa.
- For the PI/CF2000, the residual strain value of 3.9·10^−4^ mm/mm was minimal and increased by almost three times. In the PEI/CF2000 case, this parameter was higher (5.7·10^−4^ mm/m), but the increase rate was similar in the magnitude and dynamics under cyclic loads.

The fracture of the neat polymers predominantly occurred due to the development of residual strain in the LCF mode. Their sharp increase, simultaneous with the decrease in the hysteresis loop area, reflected the process of the exhaustion of their capacity for plastic straining.

In general, it is necessary to highlight two obtained results when testing at a load level close to the offset yield stress: (1) the low residual strain for the PI/CF200 composites at both cyclic load levels of 74 and 88 MPa, i.e., this parameter was not affected by increasing the load; (2) the level for the PEI/CF200 composite was commensurate with that for the neat PEI of 13·10^−4^ mm/mm.

**Figure 11:**
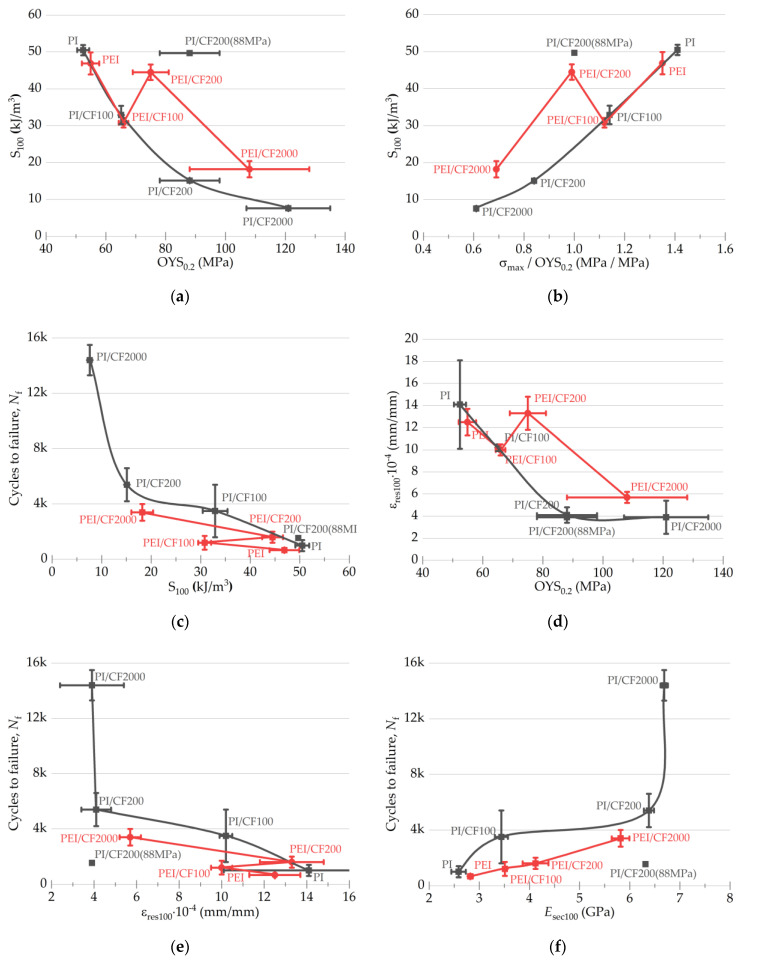
The relationships of both the elastic and plastic characteristics of the PI- and the PEI-based composites with the hysteresis loop areas *S* and the residual strain values (at 100 cycle): (**a**) *S*_100_ = *f*(OYS_0.2_); (**b**) *S*_100_ = *f*(*σ_max_*/OYS_0.2_); (**c**) *Nf* = *f*(*S*_100_); (**d**) ε_res100_ = *f*(OYS_0.2_); (**e**) *Nf* = *f*(ε_res100_); (**f**) *Nf* = *f*(*E*_sec100_).

Based on these data, a number of correlation dependencies were drawn (Figure 11) to establish possible relationships between them.

Under testing conditions, the area of the hysteresis loops *S* varied in a proportion to the offset yield stress, and this trend was generally similar for both types of the studied polymers (Figure 11a). However, it increased abruptly for the composites loaded with SCFs of 200 µm long, regardless of the polymer matrix type, under cyclic loading at the offset yield stress level. A similar trend can be observed in Figure 11b, showing the *σ_max_*/OYS_0.2_ = *f*(*S* at 100 cycles) dependences.

Searching for any correlations between the parameters of the mechanical hysteresis loops and the fatigue life (Figure 11c) also revealed a step-like change in the *N*_f_ value when tested at the offset yield stress level. Nevertheless, in general, greater durability values were observed with the larger loop areas for the PI-based composites.

**Figure 12:**
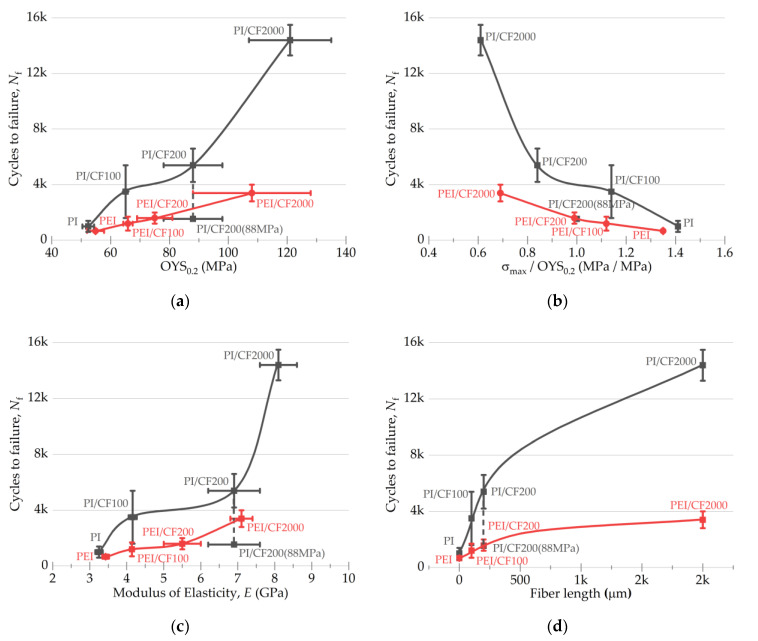
The dependences of a number of cycles to a failure on the elastic and plastic characteristics of the PI- and PEI-based composites (**a**–**c**), as well as the lengths of SCFs (**d**).

Concluding the section devoted to the search for the correlation dependences in the fatigue tests, the authors present some more curves for both types of composites (Figure 12). The conclusion is that the cyclic durability of the PI-based composites was greater after loading with SCFs of all studied types. In addition, the durability of the composites based on both matrix types expectedly increased by increasing their offset yield stress (Figure 12a), the elastic modulus levels (Figure 12c), and the lengths of SCFs (Figure 12d). The authors also noted that the range of cyclic durability values was lower for the PEI matrix.

The revealed features of the strain behavior under fatigue test conditions were further analyzed from the standpoint of changes in the structural characteristics, namely XRD data.

#### 3.4.4. XRD Spectra

**Figure 13:**
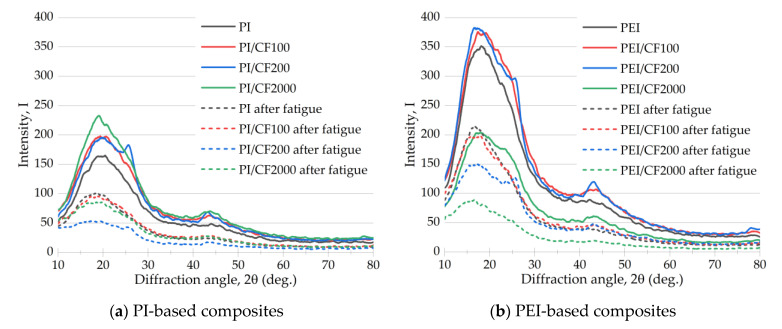
The X-ray diffraction (XRD) spectra of the PI- (**a**) and PEI-based (**b**) composites before and after the fatigue tests.

Figure 13 shows XRD spectra for the neat PI and PEI, as well as their composites before and after the fatigue tests. To ensure the identity of the XRD data, the spectra were obtained on the same specimens, but the focus was on their heads (outside the clamping zone in a hydraulic gripper; assuming that the strain development there did not occur at all), as well as on the gauge length. The following characteristic patterns should be noted:

1. Since the studied materials belonged to the amorphous class, any pronounced peaks did not appear on the spectrograms.
2. Despite the fact that the reflection intensity in the XRD spectra was plotted in relative units, the intensity of the main (broadened) maximum was more than two times higher for PEI than for PI (170 and 350 a.u., respectively).
3. After the fatigue tests, the intensity of the main maximum in the XRD spectrum significantly decreased for all tested specimens, both neat polymers and their composites.

Despite the obvious difference in the quantitative values of the intensity of the main maxima in the XRD spectrum of the PI and PEI composites with different types of SCFs, the authors preferred not to discuss these results from the standpoint of the revealed variations. This was due to both the specifics of measuring this parameter in arbitrary units and the necessity of obtaining a large amount of statistical data (considering the possible dispersion of the fatigue test results).

Thus, two key conclusions based on the XRD analysis results should be considered:

1. Greater “orderliness” of the amorphous structure of both the neat PEI and its composites, which may be the reason for the lower dispersion in the fatigue life for the PEI-based specimens.
2. A significant decrease in the intensity of the main maximum in the XRD spectrum as a result of the fatigue tests, which indicated a change in the internal structure of both polymers and their composites. This was primarily due to the formation of microscopic damage. This result was very relevant, since such scattered damage was small and almost impossible to observe with available methods of non-destructive testing.

## 4. Discussion

As noted above, the main goal in the development of PEI (unlike its predecessor, PI) was to facilitate processing by conventional thermoplastic methods [50,51,52]. Such procedures were carried out at temperatures above the melting point, as a rule, and relatively low levels of loads on the molten polymer. Therefore, the flexibility of the polymer chain, achieved by loading with hinged oxygen atoms [53,54], along with the PEI molecular weight [55], played a critical role in ensuring the processability of both neat PEI and its composites. The downside of the discussed chemical structure of PEI was its lower resistance to oxidation compared to PI [56].

On the other hand, some authors discussed the effect of the PEI matrix structure on adhesion to modifying (polymeric) fillers and reported the possibility of solving it by chemical methods [57]. At the same time, the similarity of PEI with other types of HPP prompted a significant number of studies focused on the design of polymer–polymer composites (blends); for example, PEEK–PEI composites [58,59]. However, the most common research was focused on the design of fiber-reinforced composites, where adhesion issues play a key role [60].

To date, some “reference” data on the fatigue life of particulate both PI- and PEI-based composites have been published [4], but they were obtained for industrially produced grades and did not focus on the reasons for the variations in the fatigue behavior, primarily when using different types of matrices.

In the present study, both the PI- and PEI-based composites showed very similar mechanical and markedly different fatigue properties with varying lengths of SCFs. Most likely, the closeness in the pattern of their strain behavior was associated with the amorphous structure of the polymers, as well as a certain level of the mechanical properties of the composites during their dispersed filling with reinforcing SCFs. The aspect of adhesion should have an impact, but according to the SEM data, it could be ignored as paramount.

In contrast, both types of composites showed pronounced differences in fatigue properties. The authors stated that the variations in the flexibility of the polymer chains of the PI and PEI could be considered one of the determining factors. As an indirect confirmation, the authors considered it appropriate to refer to a previous paper reporting the tribological properties of PI/10CCF/10PTFE and PEI/10CCF/10PTFE composites [61]. It was shown that the PEI-based composite was characterized by a two-times-lower friction coefficient with mechanical properties close in value under mild conditions of tribological loading.

However, the identically filled PI-based composites showed higher fatigue life values at AR = 20 and AR = 200. In the absence of strong adhesion between the matrices and SCFs (Figure 2), the loads applied resulted in the initiation and propagation of discontinuities at the interphase boundaries (the mesoscale level [62]). Loading with 10 wt.% SCFs improved the mechanical properties and the fatigue life of the composites in proportion to the length of SCFs (Figure 12, d) but did not improve the polymer’s ability to resist crack propagation. According to the authors, an increase in the content of SCFs, for example, up to 30 wt.%, should neutralize the difference between the studied thermoplastics.

**Figure 14:**
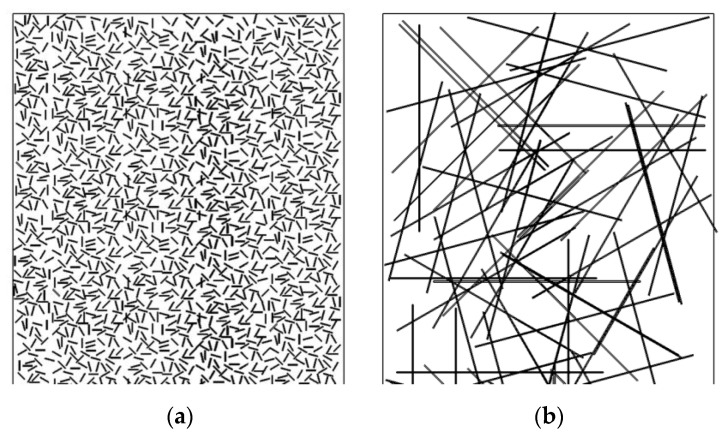
A schematic diagram of the distribution of SCFs with lengths of 100 (**a**) and 2000 (**b**) µm.

Under cyclic loads at the offset yield stress, both *E*_sec_ and *E*_dyn_ levels were significantly higher for the PEI/CF200 composite than the PEI/CF200. In the PI/CF200 case, *E*_sec_ and *E*_dyn_ values were close to that for the PI/CF2000 specimen, while they were lower for the PEI/CF200 composite and closer to that for the PEI/CF100 one. At AR = 20, SCFs reinforced the composites to a greater extent than at AR = 10 (Figure 14a). This fact was especially evident for the PI/CF200 composite due to the greater rigidity of the PI matrix.

However, the greatest difference in fatigue resistance was shown by the composites with the longest SCFs of 2000 µm. The specificity of their structure was determined by the formation of a rigid reinforcing frame (see the diagram in Figure 14), in which the role of adhesion had to be manifested to a lesser extent. In this case, the molecular structure of the binder played an important role again. At the same time, this phenomenon affected the magnitude of hysteresis losses. Figure 7 reflects the fact that the hysteresis loss was twice as high for the PEI-based composite. Both the pattern of the change and the magnitude of the residual strain did not differ much, and the dynamic and secant moduli changed in a similar way under cyclic loading. It could be assumed that it was the accumulation of scattered damage in the more adaptable composite with the more flexible polymer chain that determined its lower fatigue life.

Loading the PI with SCFs of various lengths resulted in greater improvement in the mechanical properties under both static (Figure 5) and fatigue (Figure 12d) loads compared to PEI. This may be due to the lower creep of the PI, with its greater rigidity. The more rigid PI matrix, straightening the framework of unattached SCFs, contributed to a greater extent to the effective redistribution of stress in the composites.

It should be noted that PI, being the higher temperature thermoplastic, can operate at temperatures up to 250 °C, while this threshold does not exceed 200 °C for PEI. Nevertheless, PEI is more technological, primarily due to easier processing. If the upper operating temperature is not a limitation, PEI is the clear favorite, also because of its lower cost.

Based on the results of the comparative analysis of the PI and PEI polymers with similar mechanical properties, some recommendations can be suggested for their application. When temperature operation conditions are decisive, the more temperature-resistant PI should be chosen. It retains its properties up to 250 °C, while the temperature should not exceed 200 °C in the PEI case. At the same time, the PI processing is more complex due to the higher temperatures and a significantly lower melt flow rate. However, the mechanical properties of the PI-based composites are greater, including the durability under cyclic loading (in the LSF mode). Therefore, the PI is recommended for use in more critical products, where the complexity and the cost of the technological routes (including processing temperatures and pressures) can be justified.

## 5. Conclusions

The analysis of the research data enabled us to identify the key results and patterns in the fatigue behavior of the neat PI and PEI, as well as their composites, with different lengths of reinforcing SCFs.

1. The resistance to fatigue cracks of the neat PI and PEI, as well as their composites, determined their fatigue life to a minimum level (even at a reinforcing frame of SCFs 2000 µm long).
2. Unlike the PEI, the PI was less prone to the development of creep processes, probably because of the greater rigidity of the polymer molecules, which was consistent with the data of the Raman spectra, which revealed the absence of any characteristic peaks in the PI (due to its non-polar nature and the lack of hinged oxygen atoms).
3. The monotonous decrease in the normalized value of the dynamic modulus for the PEI-based composite indicated the continuous formation of scattered damage, while such a change began in the PI-based specimens at a value of at least 50% from its fracture point.
4. In the case of SCFs 2000 µm long, the length of SCFs was comparable to the specimen thickness, causing the formation of a spatial framework of unattached SCFs at AR = 200. The lower rigidity of the polymer matrix provided more effective resistance to the accumulation of scattered damage with the simultaneous higher creep resistance. Under such conditions, the adhesion factor had a lesser effect.
5. The determining role of the accumulation of cyclic damage in the neat PI and PEI, as well as their composites reinforced with SCFs, was confirmed by the results of the XRD spectra analysis.

The authors noted that all materials (both the neat polymers and their composites) were tested at the same load level, which was both significantly above and below the offset yield stresses. At the same time, the offset yield stresses (and the elastic modulus) could differ for identical types of reinforcing fibers (SCFs of the same type and length). In the PI/CF200 case, increasing the maximum cycle load up to 88 MPa resulted in similarities both in the loop area and the fatigue life compared to the PEI/CF200 composite. On the other hand, the residual strain was significantly lower for the PI/CF200 specimen than the PEI/CF200. Thus, the fatigue life of the composites was determined not only by the molecular structure of the matrix but also by their offset yield stress.

## Figures and Tables

**Table 1:** Key physical and chemical properties of PI and PEI.

Brand, Manufacturer	Density (25 °C), g/cm^3^	Rockwell Hardness (M)	Glass Transition Temperature (Nitrogen Atmosphere), °C	Linear Expansion Coefficient of (23–30 °C), 1/°C	Oxygen Index (LOI), %
PI SolverPI-Powder 1600	1.38	110	260	3.68 ⋅ 10^−5^	46
PEI ULTEM R00H	1.27	109	217	5.2 ⋅ 10^−5^	47

**Table 2:** The fillers for the PI- and PEI-based composites.

Brand, Manufacturer	Precursor	Length, µm	Aspect Ratio (AR)	Elastic Modulus, GPa	Ultimate Tensile Strength (UTS), MPa	Designation
UVI-12, MPRI NAS, Gomel, Belarus	Viscose	100	10	60	1200	PI/CF100 and PEI/CF100
Tenax®-A, Teijin Carbon Europe Gmbh	PAN	200	20	200	2600	PI/CF200 and PEI/CF200
Tenax®-A, Teijin Carbon Europe Gmbh	PAN	2000	200	200	2600	PI/CF2000 and PEI/CF2000

**Table 3:** The mechanical properties of the PI and PEI-based composites loaded with SCFs of various lengths.

		Ultimate Tensile Strength, UTS (MPa)	Young Modulus, *E* (GPa)	Strain at Failure, ε_f_ (%)	Offset Yield Stress, OYS_0.2_ (MPa)	Fatigue Durability, *N_f_*
	Matrix	PI/PEI	PI/PEI	PI/PEI	PI/PEI	PI/PEI
Filler						
0		107.0 ± 5.0/	3.35 ± 0.1 /	8 ± 2.2 /	52.4 ± 2 /	1000 ± 400 /
		112.0 ± 3.0	3.4 ± 0.1	6.6 ± 1.6	54.9 ± 2.9	670 ± 40
10 wt.% CF100		119.2 ± 0.9.0 /	4.2 ± 0.1 /	5.6 ± 0.1/	65.1 ± 0.6 /	3500 ± 1900 /
		102.0 ± 10.0	4.1 ± 0.1	3.7 ± 0.8	65.9 ± 1.6	1200 ± 500
10 wt.% CF200		111.0 ± 10.0 /	6.9 ± 0.7 /	2.3 ± 0.03 /	88.0 ± 10.0 /	5400 ± 1200 /
		104.5 ± 1.6	5.5 ± 0.5	3.2 ± 0.7	75.0 ± 6.0	1600 ± 400
10 wt.% CF2000		128.0 ± 6.0 /	8.1 ± 0.5 /	1.9 ± 0.1 /	121.0 ± 14.0 /	14,400 ± 1100 /
		120.0 ± 10.0	7.1 ± 0.3	2.1 ± 0.1	108.0 ± 20.0	3400 ± 600

(± is the standard deviation of the measurements)

## Data Availability

Not applicable.

## Supplementary Materials

The following supporting information can be downloaded at: https://www.mdpi.com/article/10.3390/polym15051228/s1, Figure S1: The SEM micrographs of fillers loaded in the PI- and PEI-based composites; Figure S2: The FTIR spectra for the neat PI and PI-based composites before and after the static and fatigue tests; Figure S3: The FTIR spectra for the neat PEI and PEI-based composites before and after the static and fatigue tests.
