# Supplementary material for: Effect of Polymer Matrix on Inelastic Strain Development in PI- and PEI-Based Composites Reinforced with Short Carbon Fibers under Low-Cyclic Fatigue
Source: Polymers (Basel). 2023 Feb 28;15(5):1228. doi: 10.3390/polym15051228 (PMC10007074; doi:10.3390/polym15051228)
Supplement: Supplementary file 1 [file polymers-15-01228-s001.zip › polymers-2234819-supplementary.pdf]

polymers

MDPI

Article

Effect of Polymer Matrix on Inelastic Strain Development in PI- and PEI-Based Composites Reinforced with Short Carbon Fibers under Low-Cyclic Fatigue

Sergey V. Panin [1,2,*], Alexey A. Bogdanov [1,2], Alexander V. Eremin [1], Dmitry G. Buslovich [3], Ivan S. Shilko [4]

Supplementary materials

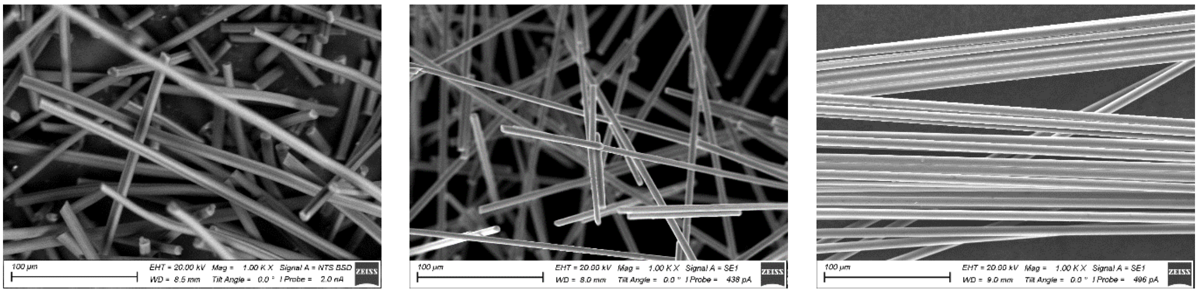

(**a**) (**b**) (**c**)

Figure S1. The SEM micrographs of fillers loaded in the PI- and PEI-based composites: "UVI-12 100μm" (**a**); "Tenax®-A 200μm" (**b**); "Tenax®-A 2000μm" (**c**).

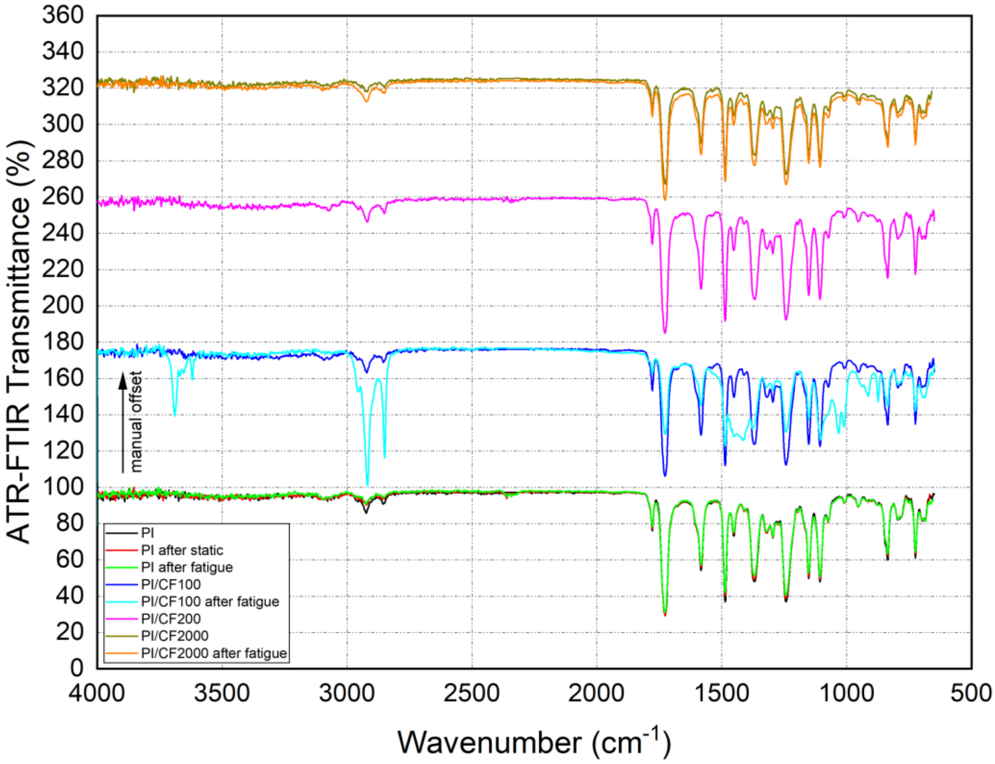

Figure S2. The FTIR spectra for the neat PI and PI-based composites before and after the static and fatigue tests.

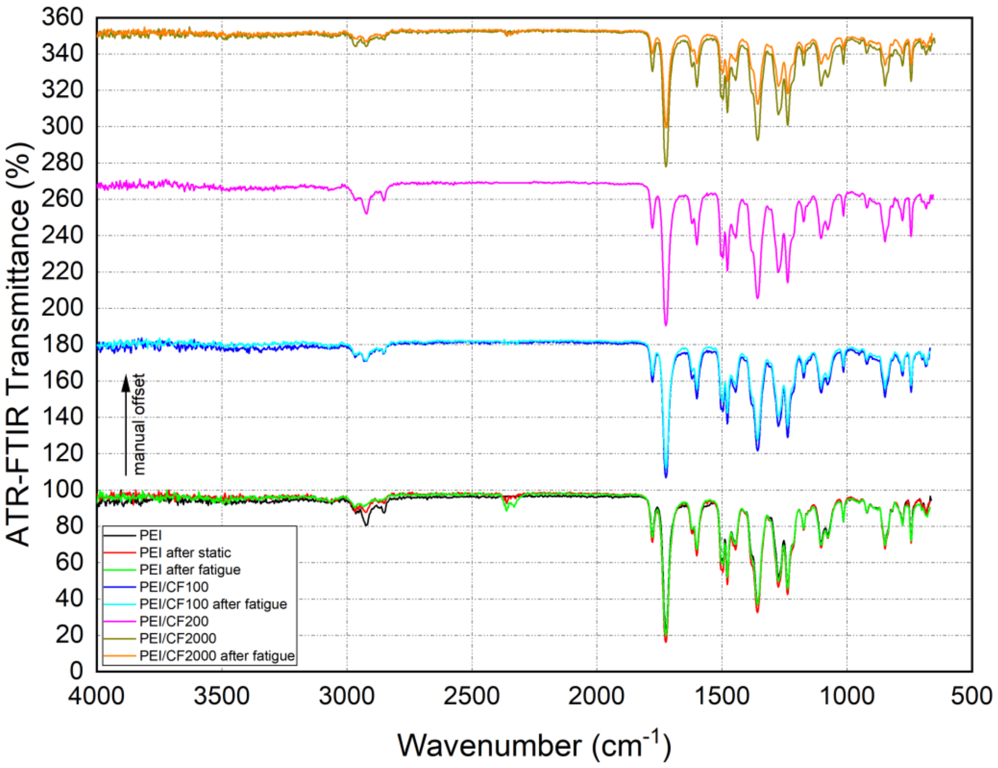

Figure S3. The FTIR spectra for the neat PEI and PEI-based composites before and after the static and fatigue tests.
